# Supplementary figures and images for: Integrative host transcriptomic and mucosal microbiome profiling reveals region-specific host-microbiome associations across the human intestine
Source: bioRxiv. 2026 May 14:2026.05.13.725025. Preprint. [Version 1] doi: 10.64898/2026.05.13.725025 (PMC13192753; doi:10.64898/2026.05.13.725025)

**A****Faith's PD**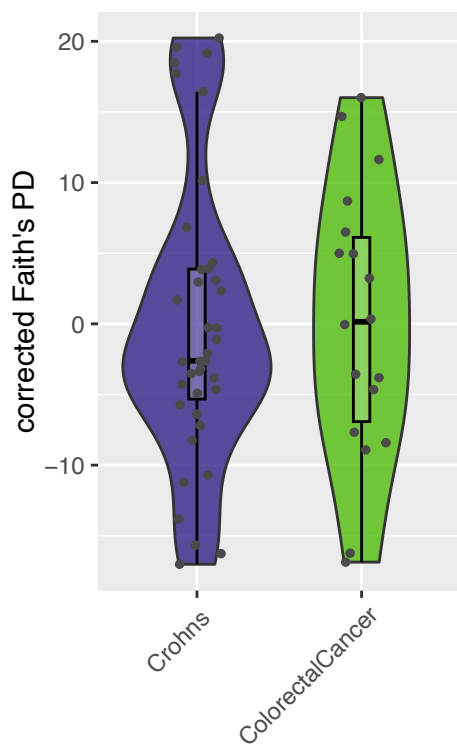**Shannon**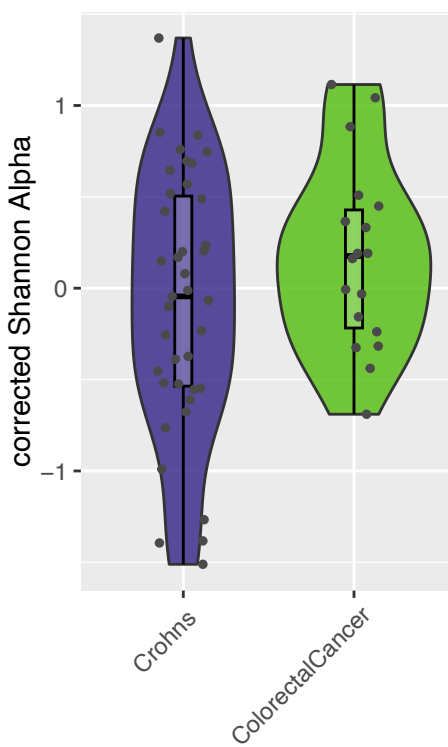**Richness**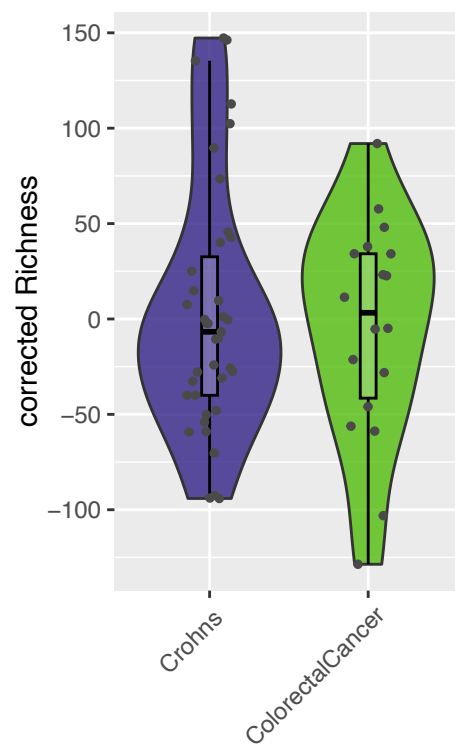**B**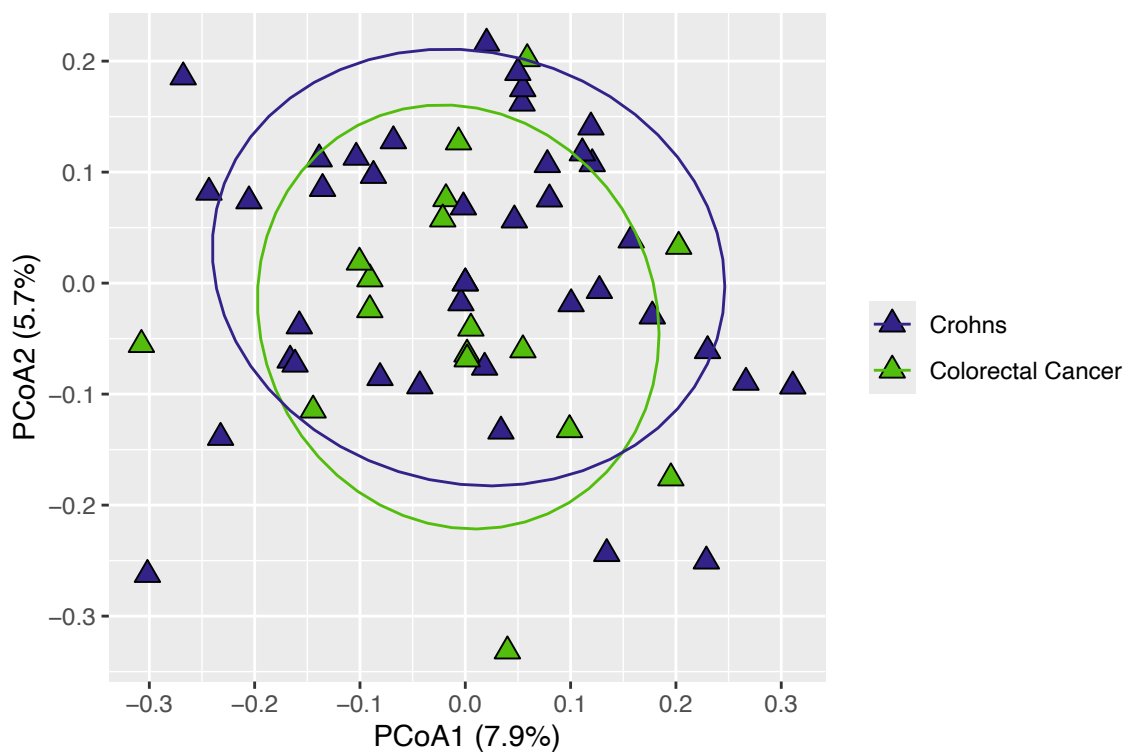

Supplement: Supplement 1 — A) Covariate-corrected Faith’s phylogenetic diversity (left), Shannon alpha diversity (middle), and richness (right) of right colon samples across all individuals, grouped by underlying disease. No significant difference was detected across disease groups for richness, Shannon alpha diversity, and Faith’s phylogenetic diversity (p > 0.05 for all, ANOVA), though statistical power was limited by the modest number of colorectal cancer samples. B) Microbiome composition did not vary significantly with underlying disease (p = 0.28, PERMANOVA). The PCoA plot shows individuals projected based on Bray–Curtis distance and colored by underlying disease. If underlying systemic inflammation were substantially affecting the mucosal microbiome in these macroscopically non-inflamed samples, we would expect to detect diversity or compositional differences between groups. The absence of such differences is consistent with the interpretation that large-scale inflammation-driven microbiome disruption is unlikely in our cohort, though we cannot exclude subtler effects. [file media-1.pdf]

**A**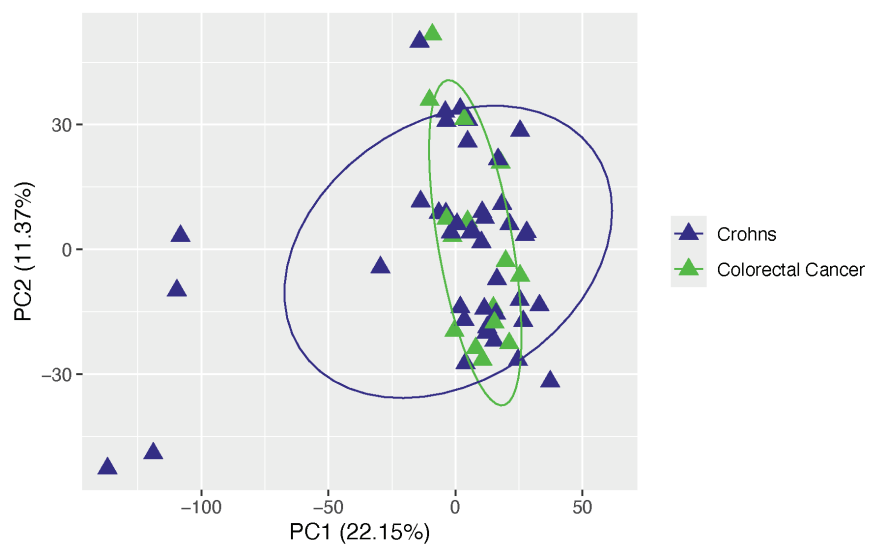**B**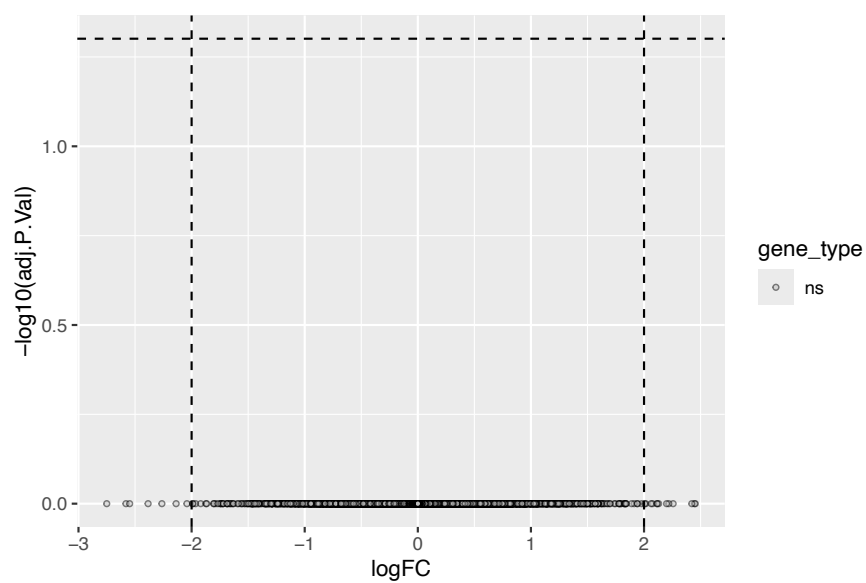

Supplement: Supplement 2 — A) PCA plot shows right colon samples colored by underlying disease, with Crohn’s samples in blue and colorectal cancer samples in green. B) Volcano plot showing that there were no significantly differentially expressed genes based on underlying disease. Log fold changes (logFC) > 2 and adjusted p-values < 0.05 were considered significant. If underlying systemic inflammation were substantially affecting the host transcriptome between groups, we would expect to see gene expression profile differences between groups. The absence of such differences is consistent with the interpretation that large-scale inflammation-driven gene expression disruption is unlikely in our cohort, though we cannot exclude subtler effects. [file media-2.pdf]

Microbe canonical variate

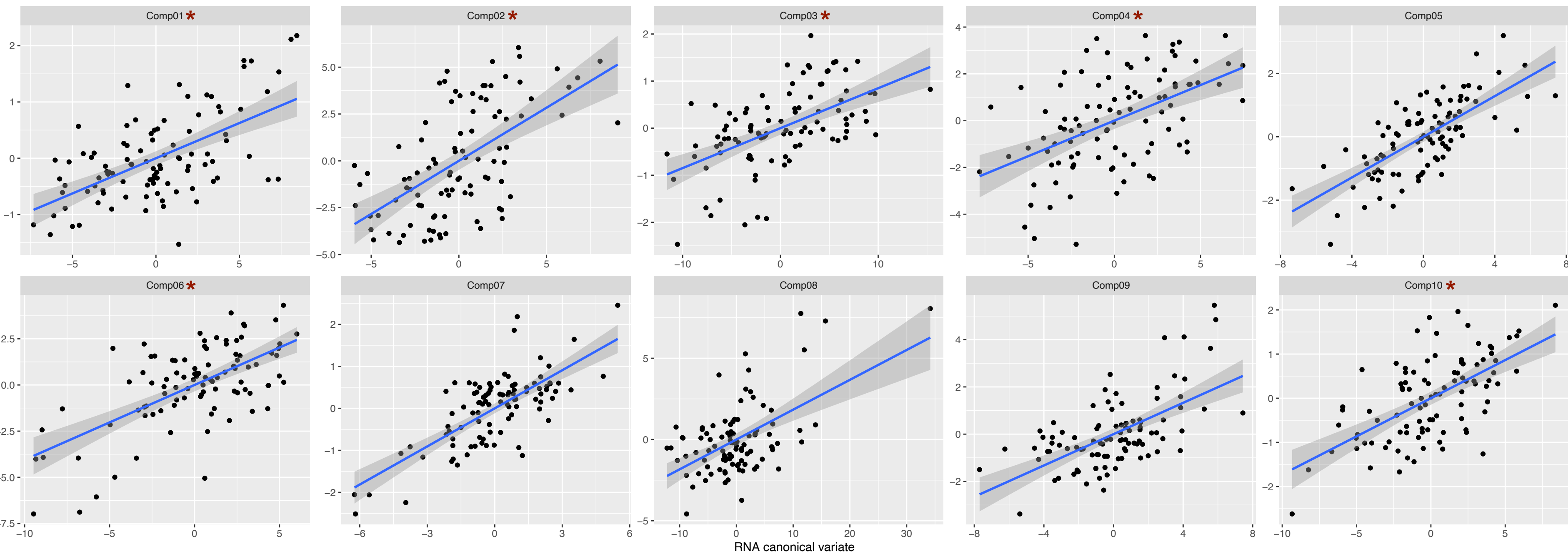

Supplement: Supplement 3 — Top 10 sparse CCA components for the intestine-wide analysis. Points in each plot represent the canonical variate scores contributing to each individual. Components 1, 2, 3, 4, 6, and 10 (marked with a red asterisk) were significantly correlated after multiple test correction (adjusted p < 0.1, rho > 0.49, Pearson correlation). [file media-3.pdf]

## Terminal ileum

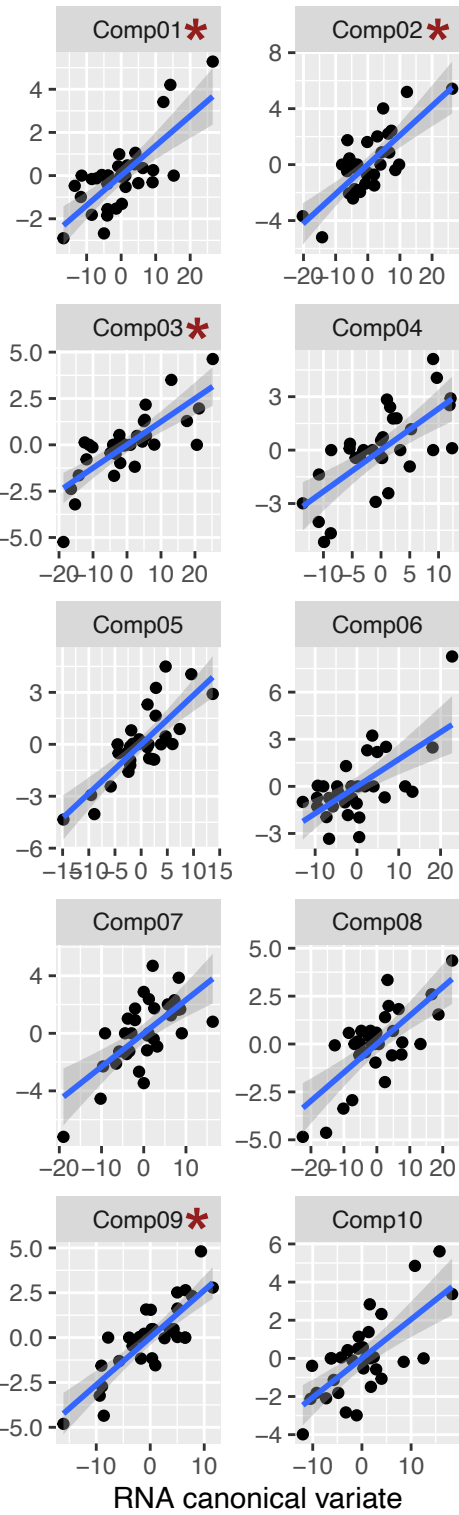

## Cecum

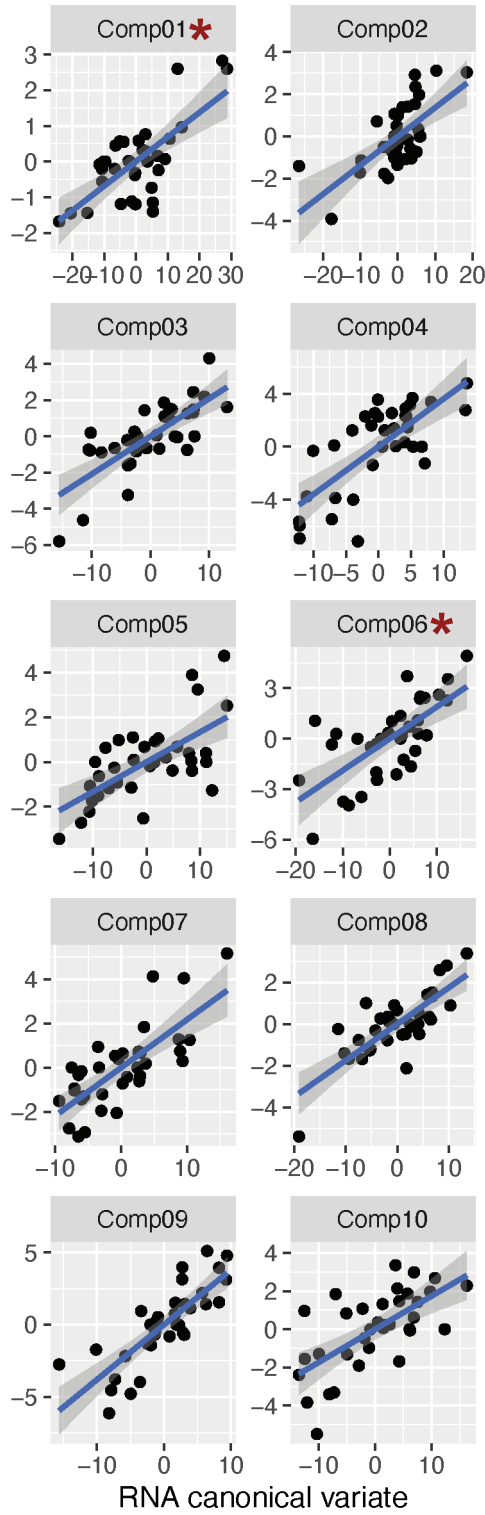

## Right colon

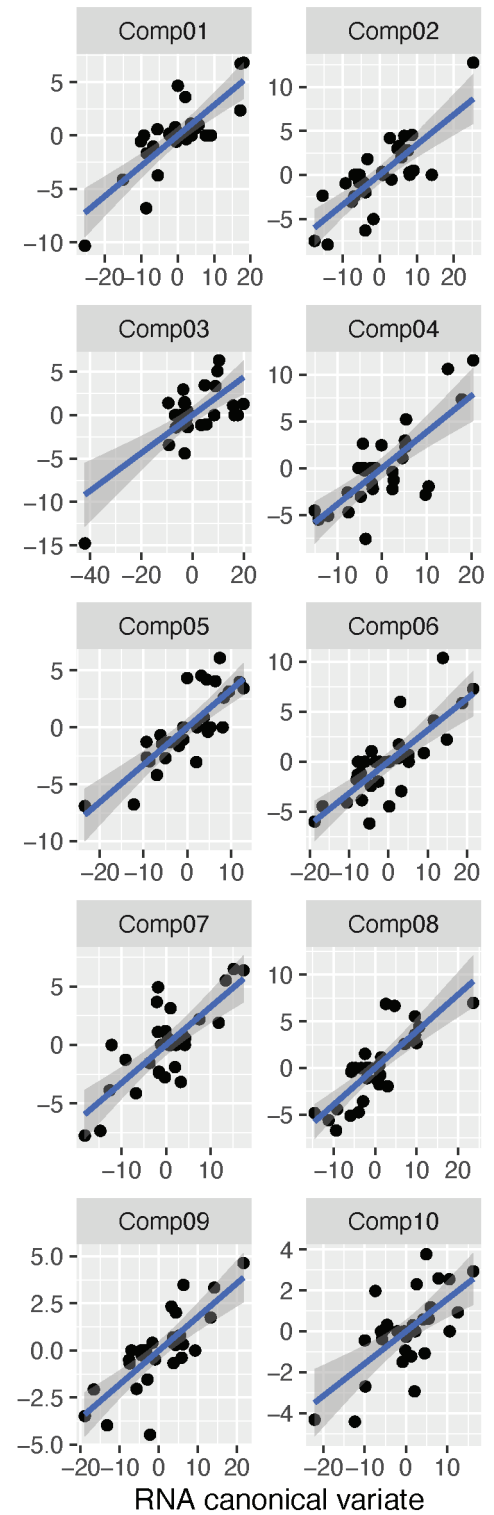

Supplement: Supplement 4 — Top 10 sparse CCA components for each region. Locations are in order from proximal to distal, left to right. Points in each plot represent the canonical variate scores contributing to each individual. Components 1, 2, 3, and 9 for the terminal ileum and components 1 and 6 for the cecum (marked with a red asterisk) were significant after multiple test correction (adjusted p < 0.1, rho > 0.71, Pearson correlation). [file media-4.pdf]

Proportion of Retained Reads

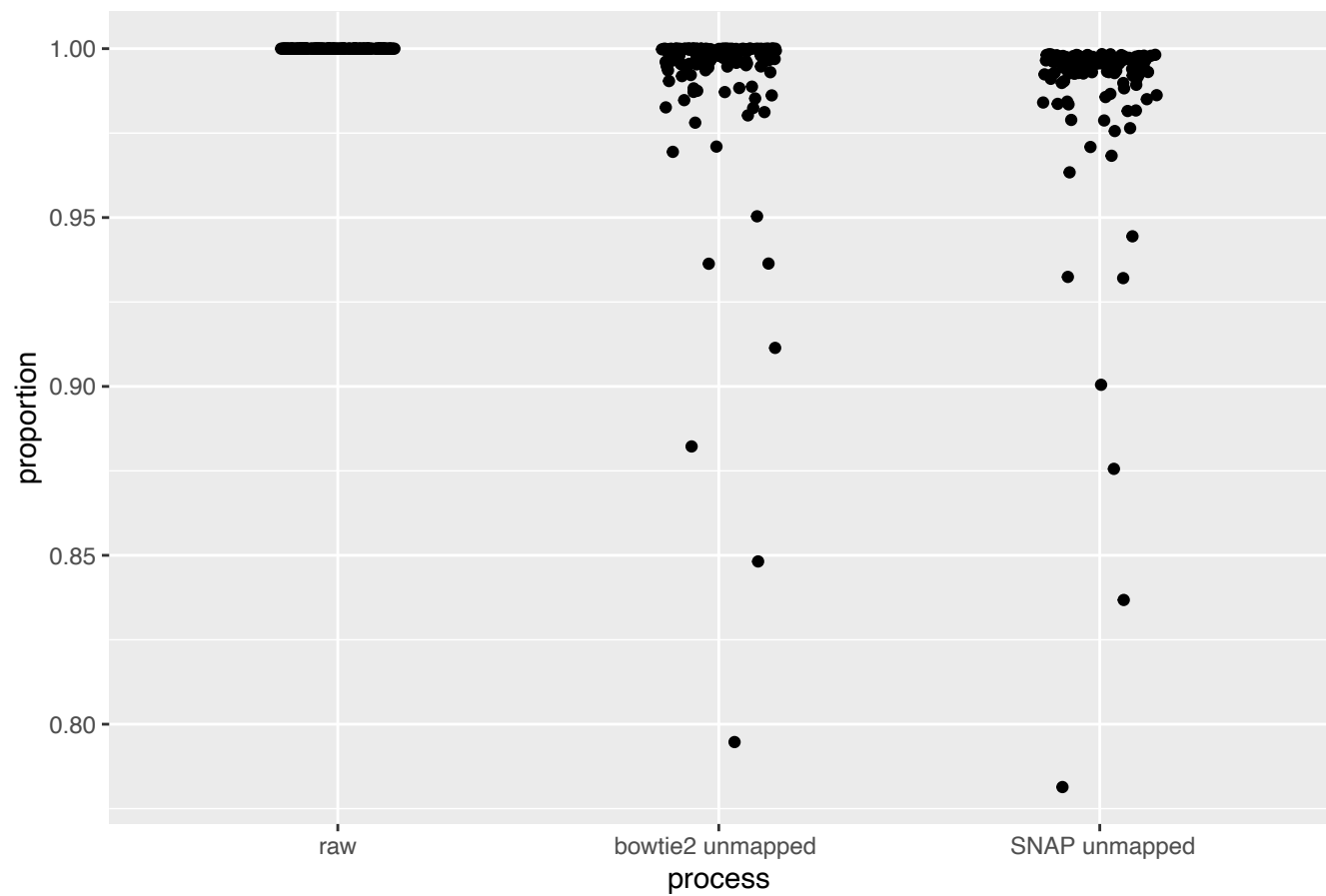

Supplement: Supplement 5 — Proportion of sequenced reads retained after each step of host decontamination compared to the starting read depth. [file media-5.pdf]

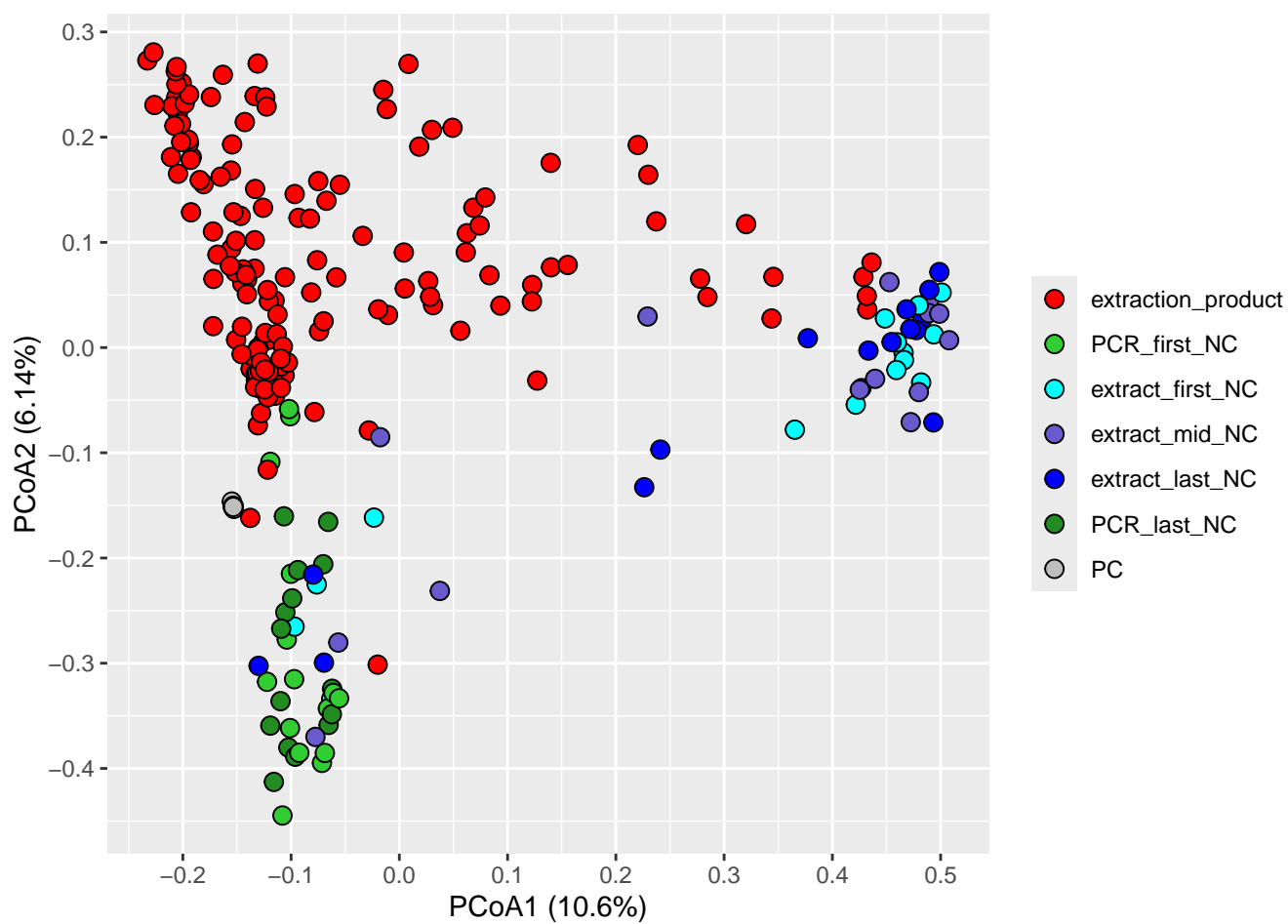

Supplement: Supplement 6 — Microbiome composition of the negative control samples differed significantly from mucosal scrapings (p = 1×10^−5^, Bray–Curtis PERMANOVA). Mucosal scraping samples are labeled in red (extraction_product). Three negative controls were included in extraction batches, with one being the first sample in the batch (extract_first_NC, light blue), one in the middle (extract_mid_NC, medium blue), and one being the last sample in the batch (extract_last_NC, dark blue). Two negative controls were included during PCR, with one being the first sample in the batch (PCR_first_NC, light green) and one being the last sample of the batch (PCR_last_NC, dark green). Zymo Microbial DNA Standard was included as a positive control during PCR (PC, gray). The clear separation between negative controls and mucosal scrapings was consistent with the interpretation that microbiome profiles in our samples reflect true biological signal rather than environmental or reagent contamination, supporting the validity of downstream analyses despite the low-biomass nature of these samples. [file media-6.pdf]

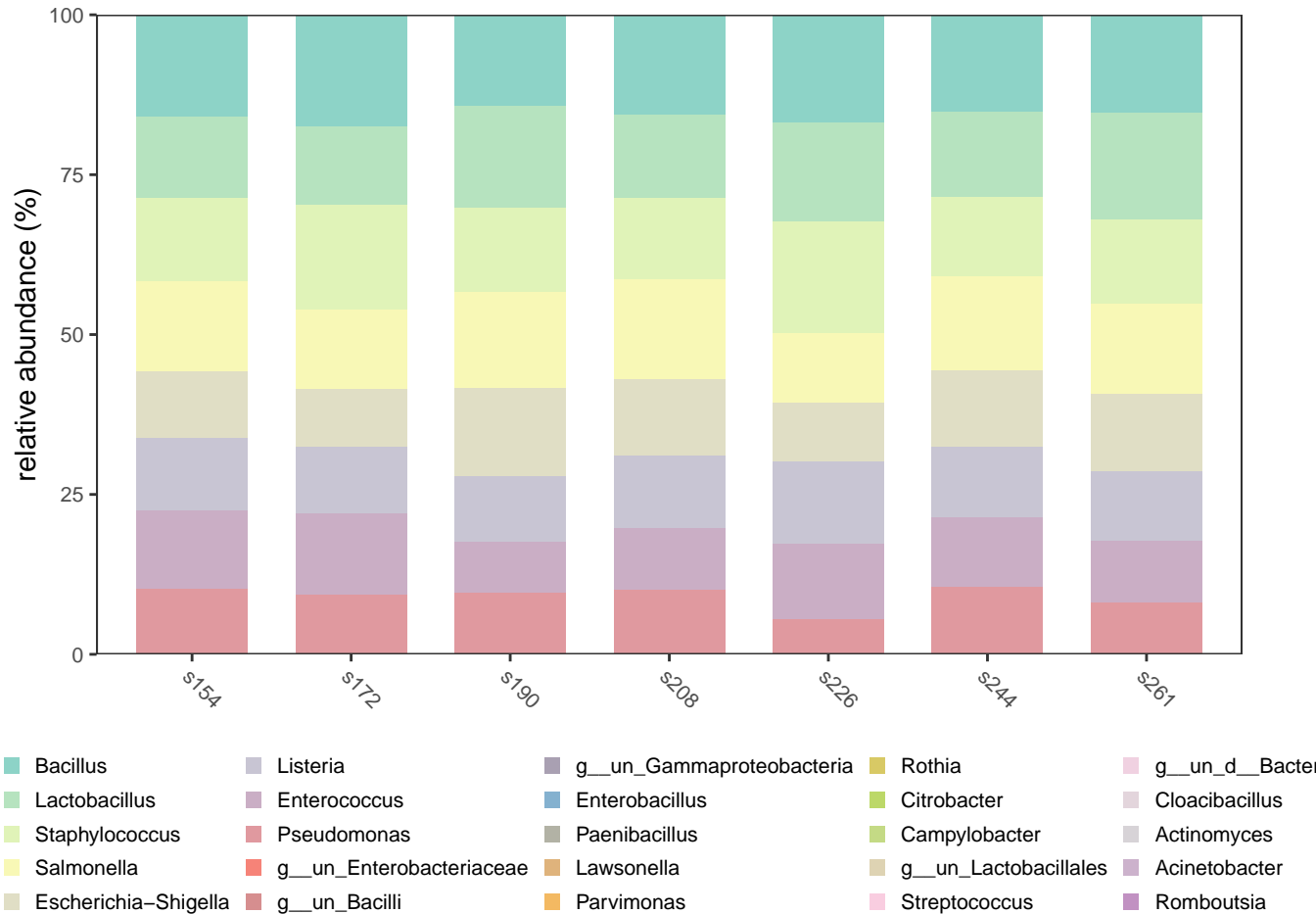

Supplement: Supplement 7 — Taxa relative abundances for the sequenced Zymo Microbial DNA Standard positive controls included in extraction batches 9–15. Positive controls were expected to include 12% Listeria monocytogenes, 12% Pseudomonas aeruginosa, 12% Bacillus subtilis, 12% Escherichia coli, 12% Salmonella enterica, 12% Lactobacillus fermentum, 12% Enterococcus faecalis, and 12% Staphylococcus aureus. Sequenced positive controls showed consistent, even distribution across expected species with minimal unexpected taxa, supporting the accuracy of our sequencing and analysis workflows and effectiveness of contamination mitigation efforts. [file media-7.pdf]

Specimen read counts

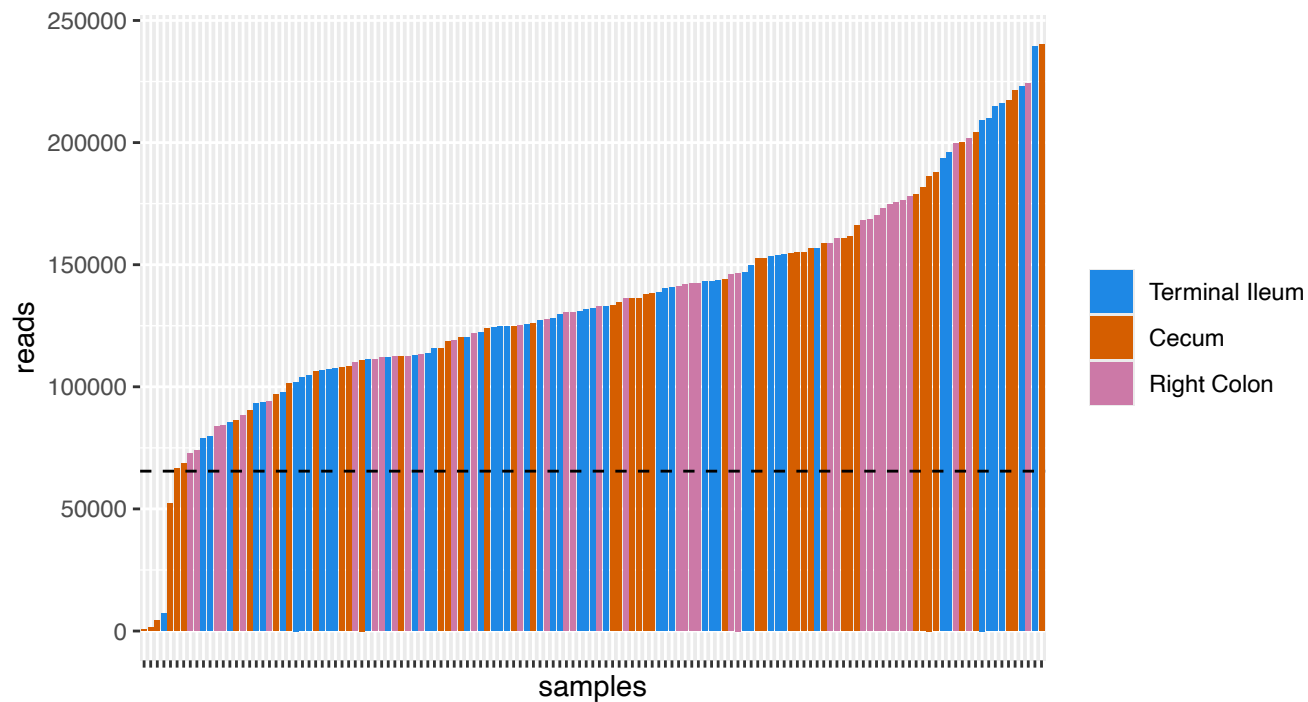

Disease read counts

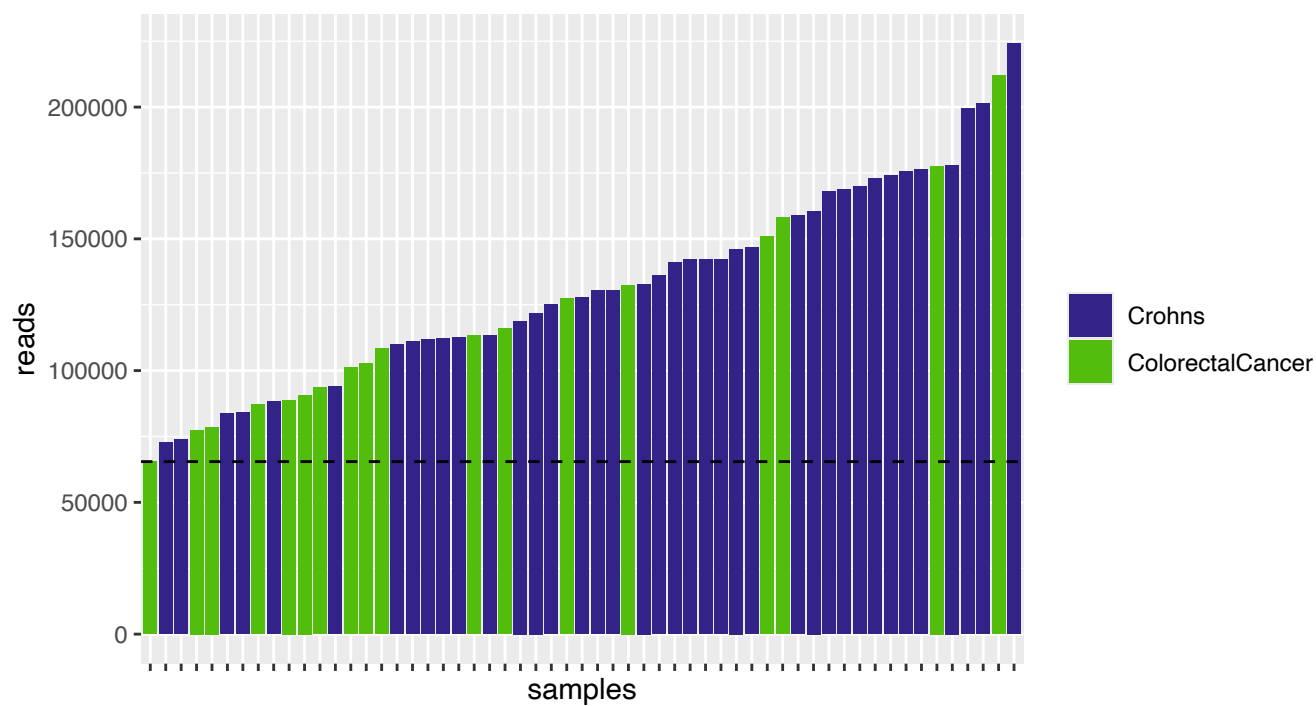

Supplement: Supplement 8 — Samples are ordered from lowest to highest read depth, colored by intestinal location (top) or underlying disease (bottom). The black dotted line indicates selected rarefaction depth. [file media-8.pdf]
